# A surge of pediatric hepatitis: Why SARS-CoV-2 should be considered guilty until proven innocent

**DOI:** 10.1016/j.amsu.2022.103956

**Published:** 2022-06-08

**Authors:** Maheen Nazir, Abia Shahid, Huzaifa Ahmad Cheema, Mohammad Yasir Essar

**Affiliations:** Department of Medicine, Rawalpindi Medical University, Rawalpindi, Pakistan; Gastroenterology and Liver Clinic, Department of Medicine, King Edward Medical University, Lahore, Pakistan; Kabul University of Medical Sciences, Kabul, Afghanistan

To The Editor,

Recently, there has been a concerning rise in acute pediatric hepatitis cases around the world. A report from the CDC portrayed a possible association between adenovirus infection and the rise in pediatric hepatitis. RT-PCR and serological testing for SARS-CoV-2 were not carried out, but it was stated that none of the previously healthy 9 children had contracted COVID-19 before [1].

Adenovirus is implicated in self-limited upper respiratory tract, conjunctival, and gastrointestinal infection in immunocompetent children. Additionally, adenovirus is known to cause hepatitis in immunocompromised patients. In children, the virus persists latently in 30% of the cases for months to years [2]. Therefore, it is understandable why it was considered to play a role in the abrupt increase in cases. But was this role strong, and more importantly, conclusive enough to overlook the possibility that SARS-CoV-2 could be responsible? Viral inclusions in hepatocytes are an established diagnostic criterion for adenoviral hepatitis [3]. However, no such inclusions were observed upon immunohistochemistry and electron microscopy in the CDC report [1].

SARS-CoV-2 tropism for hepatocytes, its propensity to cause hepatitis, and SARS-CoV-2 positivity upon RT-PCR in children with acute hepatitis make SARS-COV-2 one of the top culprits in such cases [4]. In one case report, a three-year-old female with a history of SARS-CoV-2 infection developed type 2 autoimmune hepatitis [5]. An autoimmune/inflammatory cause is more likely to respond to steroid therapy [6]. In contrast, the use of the anti-viral drug cidofovir against adenovirus to treat 2 of the cases in the CDC report yielded no positive outcomes and the only option was a liver transplant.

Researchers have received the stimulus to transparently assess a possible connection between SARS-CoV-2 infection and acute pediatric hepatitis. Amidst the emergence of new variants of concern, a preprint from India identified 37 children with COVID-19 associated hepatitis. All 37 children tested positive for SARS-CoV-2 antibodies and were managed via steroids and supportive treatment [7]. Future research should address both causation and treatment outcomes to evaluate acute pediatric hepatitis in the context of the ongoing COVID-19 pandemic.

## Ethical approval

N/A.

## Sources of funding

None.

## Author contribution

All authors equally contributed.

## Registration of research studies

1.Name of the registry: N/A.
2.Unique Identifying number or registration ID: N/A.
3.Hyperlink to your specific registration (must be publicly accessible and will be checked): N/A

## Guarantor

N/A.

## Consent

N/A.

## Funding

None.

## Declaration of competing interest

None declared.
